# Professor Caldwell on Animal Chemistry

**Published:** 1843-11

**Authors:** 


### THE WESTERN JOURNAL.

Vol. VIII.—No. V.

LOUISVILLE, NOVEMBER 1, 1843.

---

### PROFESSOR CALDWELL ON ANIMAL CHEMISTRY.

"Physiology Vindicated in a Critique on Liebig's Animal Chemistry," is the title of a spirited and labored pamphlet of ninety-four pages, by Professor Caldwell, which was published early in the summer. By some unaccountable accident we failed to receive our presentation-copy of the work, nor did any of the notices of it which have appeared reach us until we returned a few days since to the city. This apology we deem due to our readers for having so long neglected to introduce to them a western publication which is exciting, and is likely to excite, no little interest in scientific circles. It is a production abounding in vigorous thought and plausible argument, written in a style of clearness and elegance which commands general admiration. The chemico-physiological doctrines of Liebig have no where encountered such uncompromising hostility, as is evinced in the pages of this pamphlet. Dr. Caldwell, in truth, stands almost alone in his utter opposition to the application of chemistry to the explication of vital processes, and in this war upon what he styles "*dislocated chemistry*," he is met not only by the whole host of chemists, but by nearly every physiologist of note who has appeared since the time of John Hunter. By every physiologist of the present century whose works we have consulted, with, at most, but two exceptions, the light of chemistry is invoked, as friendly to the advancement of physiological science. Far from regarding the researches in animal chemistry as "forbidden aggressions" on the province of physiology, they have availed themselves of the facts brought to light by the analyses of the chemist, many of them admitting, that chemistry alone is competent to explain some of the functions of the animal economy. These, embracing some of the first names in our profession, the veteran author of "Physiology Vindicated," styles "pretended physiologists," and, in most emphatic language, closes his work with a call upon every friend of "sound physiology, to defend it from the *unhallowed usurpation and contaminating influence of dislocated chemistry.*" To his imagination Liebig seems "another Attila—the *curse of God*"—whose doctrines he conceives to be a "domination" over the philosophy of living organized matter to which it would be degrading to submit!

We listened, last winter, with great interest, and, we believe, without any unfavorable bias, to the lectures in which the views of Dr. Caldwell embodied in this pamphlet were set forth. We weighed whatever he had to urge, with his peculiar energy, against the theories of Liebig, as well as against animal chemistry in general, and the result was any thing but a conviction of the force of his objections. On the contrary they struck us as generally susceptible of a ready and satisfactory answer. Such an answer was essayed a few evenings after, and as from circumstances already mentioned no notice of Professor Caldwell's work has yet appeared in our Journal, we propose in an extended review of it to present the facts and explanations embraced in that reply, in our next number. Meantime, we would recommend "Physiology Vindicated" to the attentive perusal of all who are curious to see what of fact or argument can be opposed to animal chemistry. Y.

